# Proficiency in motor imagery is linked to the lateralization of focused ERD patterns and beta PDC

**DOI:** 10.1186/s12984-025-01571-6

**Published:** 2025-02-20

**Authors:** Irma Nayeli Angulo-Sherman, Umberto León-Domínguez, Antonio Martinez-Torteya, Gilberto Andrés Fragoso-González, Mayté Verónica Martínez-Pérez

**Affiliations:** 1 Departamento de Ingeniería Biomédica, Vicerrectoría de Ciencias de la Salud, Universidad de Monterrey, Av. Ignacio Morones Prieto 4500, 66238 San Pedro Garza García, México; 2 Laboratorio de Cognición Humana y Estudios del Cerebro, Departamento de Psicología, Vicerrectoría de Ciencias de la Salud, Universidad de Monterrey, Av. Ignacio Morones Prieto 4500, 66238 San Pedro Garza García, México; 3 Escuela de Ingeniería y Tecnologías, Universidad de Monterrey, Av. Ignacio Morones Prieto 4500, 66238 San Pedro Garza García, México

**Keywords:** Rehabilitation, EEG, Motor imagery, PDC

## Abstract

**Background:**

Motor imagery based brain-computer interfaces (MI-BCIs) are systems that detect the mental rehearsal of movement from brain activity signals (EEG) for controlling devices that can potentiate motor neurorehabilitation. Considering the problem that MI proficiency requires training and it is not always achieved, EEG desirable features should be investigated to propose indicators of successful MI training.

**Methods:**

Nine healthy right-handed subjects trained with a MI-BCI for four sessions. In each session, EEG was recorded for 30 trials that consisted of a rest and a dominant-hand MI sequence, which were used for calibrating the system. Then, the subject participated in 160 trials in which a cursor was displaced on a screen by performing MI or relaxing to hit a target. The session’s accuracy was calculated. For each trial from the calibration phase of the first session, the power spectral density (PSD) and the partial directed coherence (PDC) of the rest and MI EEG segments were obtained to estimate the event-related synchronization changes (ERS) and the connectivity patterns of the $\theta$, $\alpha$, $\beta$ and $\gamma$ bands that are associated with high BCI control (accuracy above 70% in at least one session). Finally, t-tests and rank-sum tests ($p<0.05$, with Benjamini-Hochberg correction) were used to compare the ERS/ERD and PDC values of subjects with high and low accuracy, respectively.

**Results:**

Proficient users showed greater $\alpha$ ERD on the right-hand motor cortex (left hemisphere). Furthermore, the $\beta$ PDC related to the ipsilateral motor cortex is commonly weakened during motor imagery, while the contralateral motor cortex $\gamma$ PDC is enhanced.

**Conclusions:**

Motor imagery proficiency is related to the focused and lateralized event-related $\alpha$ desynchronization patterns and the lateralization of $\beta$ and $\gamma$ PDC. Future analysis of these features could allow complimenting the information for assessment of subject-specific BCI control and the prediction of the effectiveness of motor-imagery training.

## Background

A brain-computer interface (BCI) is a system that acquires and interprets brain activity signals, usually measured using electroencephalography (EEG), to provide a new communication channel for controlling a device[1, 2]. The use of a BCI involves performing a particular cognitive event that is detected by the system, such as motor imagery (MI), i.e., mental rehearsal of movement without movement execution [3, 4].

The typical MI-BCI system relies on the detection of event-related desynchronization (ERD) or synchronization (ERS) over specific locations and frequency components based on the relation of motor activity and the attenuation of the band power (ERD) of the $\mu$ and $\beta$ rhythms on the contralateral sensorimotor region. In contrast, the band power increases (ERS) over the same region and frequency range during rest as a sign of idling or inhibition [5, 6]. Despite the $\mu$-rhythm is a different rhythm from the $\alpha$-rhythm, the former is frequently considered as an oscillation in the $\alpha$ frequency range, with a second band in the $\beta$ range (13–24 Hz) [7, 8]. Due to the overlap of the frequencies and that the specific $\mu$ rhythms modulations are not discernible in all subjects, the analysis is commonly performed in terms of the $\alpha$ and $\beta$ bands [7, 9].

The present study is aimed to explore the relation of the maximum performance that can be achieved during a MI-BCI training of four sessions with the EEG power spectral density and the partial directed coherence of the first calibration session that was obtained before using the BCI system. This approach has the purpose of identifying EEG features that occur previous to the operation of the system that can be related to the best accuracy that can be achieved during MI-BCI training.

The relation between the subject EEG characteristics and BCI accuracy is crucial because there is no universal BCI that can be used by all people. One of the main limitations of MI-based BCIs is that the level of control that can be achieved by the operator of the system depends on the subject’s ability of producing stable and detectable EEG patterns, which requires training [10]. Even after a training period using a BCI with reliable algorithms, about 20% of the users do not show strong variations of brain activity for effective control [11], which appears to be related to a wide range of subject-specific characteristics [12, 13]. This circumstance in which a person cannot operate a particular BCI design is known as “BCI illiteracy” [14], which is a technical term that is only defined as a problem of accuracy without specifically accounting for its causes or solutions. Then, it would be useful to detect BCI inoperability before the user trains with a MI-BCI for several sessions, as the system would not provide feedback that is truly representative of the brain activity if the accuracy is close to chance level and, thus, it would not enhance the learning process during the practice of MI [15]. This is particularly relevant for the case in which the BCI is used within a neurorehabilitation process, considering that it is important to prevent maladaptive plasticity to improve the outcome of the therapy [16].

There are several studies aimed to elucidate the relation between MI-BCI performance and EEG activity. In terms of bandpower analysis, it has been reported that users with proficient BCI performance show higher ERD within the $\alpha$ and $\beta$ frequency range during MI over the electrodes associated with the motor cortex and higher ERS during rest over the same location [17–19]. Based on this known phenomenon, a sensorimotor (SMR) performance predictor has been proposed, calculated as the maximum difference between the mathematical fit of the reciprocal of the frequency, which represents the noise estimation, and the power spectral density (PSD) of the Laplacian channels C3 and C4 of a two-minute recording in which the subject is relaxed with the eyes open [18]. The predictor showed a correlation of $r=0.53$ with the accuracy of one training session with a MI-BCI system that operated based on the modulation of the SMR in 80 healthy BCI-näive subjects [18]. A similar but more complete approach is discussed in [20], where performance is predicted as the relation of the weighted sum of $\alpha$ and $\beta$ relative band power from C3 and C4 and the weighted sum of $\theta$ and $\gamma$ frequencies from the same channels, based on observations in which users with better classification performance tend to exhibit low $\theta$ and $\gamma$ activity, as well as high $\alpha$ and $\beta$ activity during rest. The correlation of the performance predictor of the subjects and their accuracy from a single session was high ($r=0.72$) after discarding outlier subjects.

There seem to be less studies about the relation of BCI accuracy and functional connectivity during MI-BCI training. Within functional connectivity measures, partial directed coherence (PDC) of EEG measurements has been proposed as an efficient approach to select features for future BCI applications [21] and it has been studied during MI tasks with the objective of proposing a classifier based on PDC values from one experimental session [22]. In relation to BCI performance, $\mu$-rhythm (8–13 Hz) PDC interactions have been reported for proficient BCI subjects over centro-frontal and centro-parietal regions, whose couplings have been already related to sensorimotor activity [15, 23, 24], while the spatial distribution of PDC interactions spread out across both hemispheres for subjects with lower classification rate [22].

It should be mentioned that most studies of BCI performance use from one training session even though learning to control a BCI could require multiple sessions [25, 26], specially in the case of PDC analysis. Thus, the analysis of both PSD and PDC that with data from multiple experimental sessions could provide complimentary information to evaluate the effectiveness of BCI training.

In summary, the paper is aimed to identify those EEG signal characteristics that better discriminate between individuals with low and high BCI proficiency using data from different training sessions. This approach could be used to find reliable EEG signal characteristics to evaluate the appropriateness of a BCI design for an individual, as well as enhance the accuracy of signal classification among different imagery tasks [27]. The novelty introduced in this research is the use of PDC to find EEG characteristics that assist researchers or clinicians in predicting which individuals can benefit more swiftly and effectively from MI-BCI based activities. For instance, discriminating these variables would also allow for improved training of machine learning algorithms by reducing the number of inputs used for training [27, 28]. Therefore, by combining PSD and PDC techniques to identify significant variables for discriminating between high and low proficiency in MI-BCI tasks, it will be possible to determine those subjects who could benefit more from BCI-based techniques in applications such as motor rehabilitation.

## Methods

### Experimental set-up

#### EEG acquisition

**Fig. 1:**
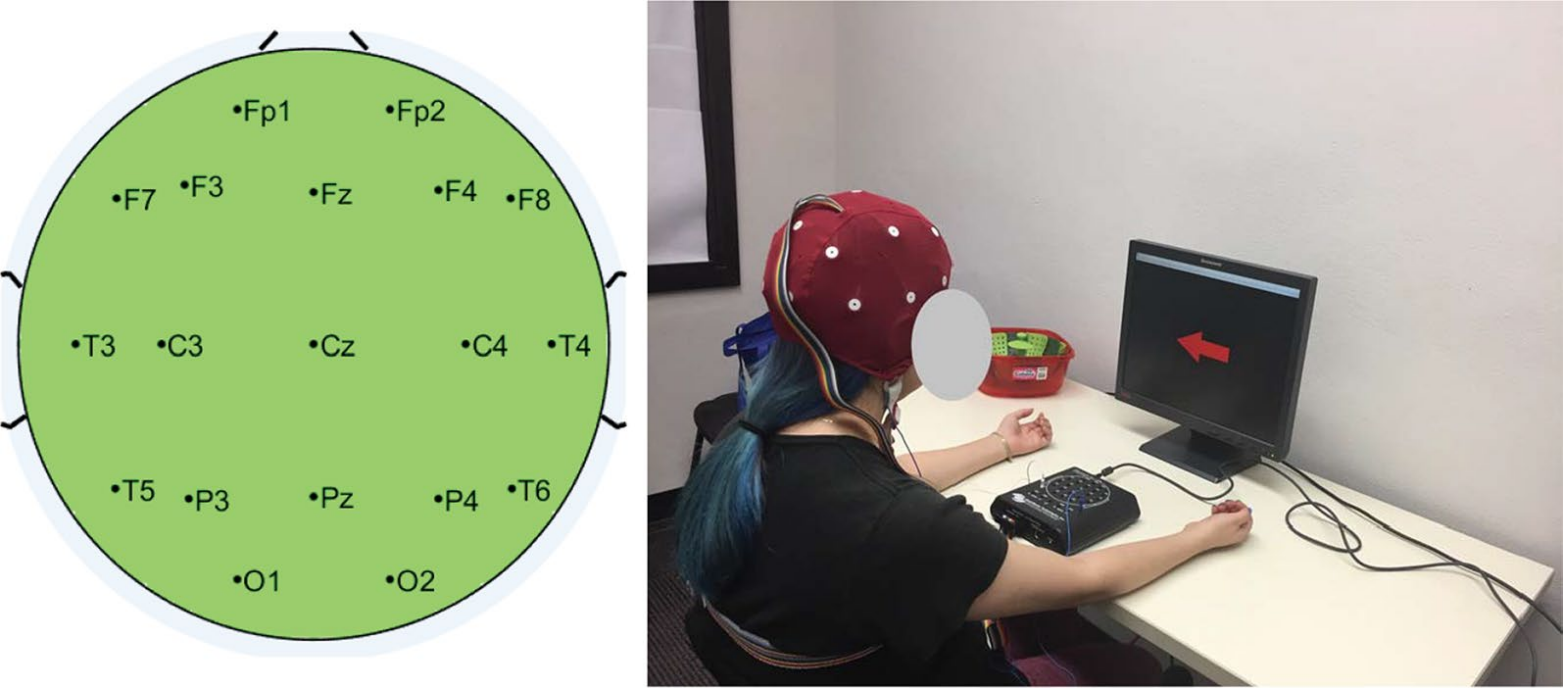
Experimental setup. EEG electrode positions (left) and a user during the experimental session (right)

A Discovery 24 system (BrainMaster Technologies, Inc.) was used to acquire the EEG at a sampling rate of 256 Hz. An Electro-Cap headset (Electro-Cap International, Inc.) was used to obtain the signals from 19 channels (FP1, FP2, F3, F4, C3, C4, P3, P4, O1, O2, F7, F8, T3, T4, T5, T6, FZ, CZ, and PZ), according to the international 10/20 system. The channels were referenced to the earlobes. The positions of each EEG sensor on the head is schematized in Fig. 1 (left). The impedance of each channel was measured at the beginning of the experimental sessions using a Checktrode 1089 (UFI) in order to verify that it was below 5 k$\Omega$. The excitation frequency that the Checktrode supplied for the impedance measurement was of 30 Hz.

In terms of software, EEG data were recorded with the help of Openvibe, which applied a fourth-order bandpass filter with cutoff frequencies at 0.1 Hz and 100 Hz and a notch filter at 60 Hz to suppress the interference of the power line. Data files were converted into a MATLAB supported format (‘.mat’) for further processing.

#### Participants

The determination of the sample size in BCI studies is limited by the difficulty of performing classical power calculations and the cost of sampling [29], which is expected to increase when the experimental protocol requires multiple experimental sessions with several trials sampled per subject. In studies as the present one, where evaluating the feasibility or adequacy of the method for assessing the change of a variable is needed before planning a larger study, it is common to perform a preliminary analysis in a small sample. In pilot studies, it has been recommended to obtain approximately 10 subjects per group (e.g., treatment) or 10% of the final study size [30].

Nine right-handed volunteers (4 male and 5 female, with an age of 20.89±1.54 years old) were recruited for this study. Each of them gave written informed consent prior to the experimental procedure, which was approved by the research ethics committee (Comité de Ética en Investigación) of the Universidad de Monterrey. None of the subjects had any reported neurological condition or metallic implants. The participants were labeled as Subject 1,2, ..., 9. The determination of hand dominance was self-reported and based on which hand is used for writing. This method was selected based on its simplicity and its correlation with hand preference for unimanual activities [31]. Besides, it has been commonly used in studies like [32] and [33].

The selected number of volunteers is comparable to other BCI studies with more than a single-day BCI experimental session and is also comparable to other preliminary EEG studies that use PDC analysis of a single session [19, 34]. Despite a small number of subjects being used, the experimental methodology seeks to characterize the individuals by acquiring enough data (i.e., trials) to obtain precise estimates for each subject for identifying the electrophysiological variables that intervene in proficiency when performing motor imagery tasks.

#### Experimental procedure

Each subject participated in four training sessions that were held on different days. The selection of four sessions was based on previous findings [35]. This number was determined to be sufficient to assess the effectiveness of a specific BCI design in healthy users under the same experimental protocol. Specifically, it aimed to evaluate whether it could increase the control level when operating the system. Thus, this protocol aims to allow the evaluation of the suitability of the training for the user. Additionally, the number of sessions was selected to reduce the effect of possible loss of motivation by keeping the number of sessions at a minimum. At least two days were left between sessions, which were scheduled within a two-week period. Each experimental session lasted approximately 1.5 h, taking into account the preparation of the EEG headset. It should be mentioned that similar inter-session intervals that leave more than one day of rest within experimental sessions have been used successfully to test the effects of neurofeedback training [35, 36].

During the experiment, the user was seated on a chair with hands resting comfortably on a table. A screen was placed in front of the participant. This screen displayed visual cues necessary for the experiment. Fig. 1 (right) shows a user performing the experiment. The volunteer was asked to avoid blinking, jaw clenching, or moving during the EEG recording.

Each session began with a ‘Stimulus Presentation’ phase in which EEG signals were obtained during left- and right-hand motor imagery and rest conditions. Next, these data were analyzed in a ‘Calibration Analysis’ phase to select subject-specific parameters that allowed the best discrimination between the dominant hand motor imagery and the rest states. These features were used to configure the BCI system for user operation. Finally, the participant trained with the BCI in a ‘Cursor Task’ that required shifting between the rest condition and the dominant-hand MI state in a control task. Meanwhile, the subject’s performance in operating the system was assessed. This protocol is entirely based on the $\mu$-rhythm tutorial of BCI2000, although it was adapted to Openvibe to achieve communication with the EEG amplifier used in this study [37].

**Fig. 2:**
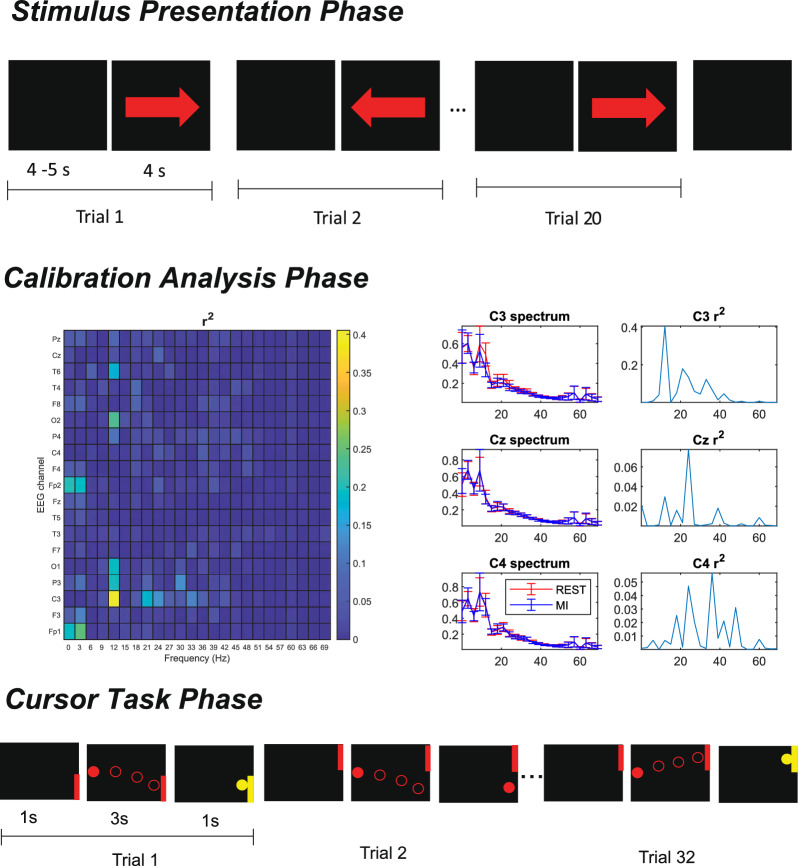
Phases of the experiment. Temporal sequence of a Stimulus Presentation run (top), Determination coefficient ($r^2$) and PSD (mean ± standard deviation) of the dominant-hand motor imagery and the rest EEG data in the Calibration Analysis (middle), and Temporal sequence of a Cursor Task run (bottom)

The procedure followed for the Stimulus Presentation, the Calibration Analysis and the Cursor Task phases are described next:

*Stimulus Presentation.* Subjects performed three runs in which EEG was recorded, with breaks of about one minute between runs. Each run consisted of 20 trials. Each run began with a label over the screen that indicated the user to be prepared for performing the upcoming instructions. Then the user had to perform a sequence of 20 trials. In each trial, an empty dark screen without a visual cue (4–5 s) indicated the user to relax and, next, an arrow pointing either to the left of right (4 s) was shown. At the end of the 20 trials, the empty dark screen was displayed again (4–5 s) before the interruption of the recording. This is schematized in Fig. 2 (top). In total, 10 trials of arrows for each direction were shown in random order in each run.

When the screen showed an arrow directed to the left or right, the user had to perform the rehearsal of opening and closing the left or right hand, respectively, without actual movement. Users were asked to recall the movement intention and the sensation that was expected to occur during the own motor execution, while avoiding any visual imagery of the movement. The same instruction was given to all subjects in an attempt to reduce the variability on the resulting brain activity, as different neural mechanisms are involved depending on the characteristics of the motor imagery task [3]. In contrast, when a dark screen showed up, the user had to relax. For this purpose, subjects were suggested to breathe calmly and focus on their breathing, as performed in other BCI studies to facilitate a relaxing rest state [38, 39].

*Calibration Analysis.* The recordings from the Stimulus Presentation phase were analyzed in MATLAB to select a user-defined frequency for the experimental session. First, the dominant-hand motor imagery and the corresponding rest EEG data were segmented for each trial, providing a total of 30 EEG segments of right-hand motor imagery and 30 of rest state for each electrode. It should be mentioned that only these segments were considered to analyze the ERS/ERD effect related to the motor imagery of the dominant hand (right), because its EEG modulation would be the one used to control de BCI. In addition, the ERS/ERD measurements are displayed relative to the power of a reference period that occurs before the event [40]. Therefore, the other fragments of the EEG signal were not required for the calibration.

The periodogram’s power spectral density (PSD) estimate was obtained for each segment (30 for motor imagery and 30 for rest) and electrode by using the “periodogram” function from MATLAB. This function approximates the periodogram as the Fourier transform of the biased estimate of the autocorrelation sequence and it allows the definition of the specific frequencies *f* for which the PSD is being calculated [41]. The formula to estimate the periodogram of a signal $x_n$ with *N* samples is:

1 $$\begin{aligned} PSD(f)= \frac{T}{N} \bigg | \sum _{n=0}^{N} x_n e^{-j2\pi fTn} \bigg |^2, \end{aligned}$$

where $-\frac{1}{2T}<f<\frac{1}{2T}$ and T is the sampling period. In particular, the PSDs were calculated with a resolution of 0.2 Hz and a range of $-$1.4 to 70.4 Hz. PSDs were divided into 24 frequency bins with a width of approximately 3 Hz, where each bin included 15 PSD evaluations to provide enough values for each bin. The first and last bins were centered at 0 Hz ($-$1.4 – 1.4 Hz) and 69 Hz (67.6 – 70.4 Hz). The width of the bin was selected in order to provide cutoff frequencies that would be used in a user-defined filter in the “Cursor Task”. Then, the mean and the standard deviation for each bin was calculated. Note that this design is based on a default configuration of the $\mu$-rhythm’s offline analysis of BCI2000.

Next, the determination coefficient $r^2$ was used for comparing the PSD distributions of motor imagery and rest conditions at each channel and frequency bin. The $r^2$-value for a specific bin and channel was calculated as [42]:

2 $$\begin{aligned} r^2= \frac{s^2_{gy}}{s^2_{g}s^2_{y}}, \end{aligned}$$

where $s^2_{gy}$ is the covariance between the conditions (*g*) and PSD observations from the selected bin and channel (*y*), while $s^2_{g}$ and $s^2_{y}$ are the variances of the condition and the PSD observations from the same bin and channel, respectively [35]. It can be noted that the formula requires that the categorical variable of “condition” has a value per observation. Dummy variables can be used in the health field to divide the data in categories or classes for performing the $r^2$ analysis [43], so a value of -1 and 1 were assigned for the for the dominant-hand motor imagery and rest, respectively. This is also described in the $\mu$-rhythm’s offline analysis of BCI2000).

$r^2$ represents the portion of variation of the PSD at a particular location and frequency that can be explained by the change of tasks (motor imagery and rest), and it ranges from 0 to 1 [44]. Therefore, the higher $r^2$-value was selected for configuring a BCI system that allowed control by identifying MI and rest conditions. It should be mentioned that the selected feature had to be consistent with the sensorimotor properties, i.e., the activity occurred on the contralateral motor cortex electrode (C3 for right-hand motor tasks) and the spectrum was attenuated during motor imagery within the 8–30 Hz frequency range. This means that the selected feature of the experimental session was the central frequency of the bin that (1) was within 8–30 Hz, (2) had the highest $r^2$-value in C3 and (3) displayed in the PSD an attenuation during MI compared to the PSD at rest. This selection was performed with visual aids of the spectral features and $r^2$. For example, Fig. 2 (middle) shows the $r^2$ channels for each channel and frequency of a user (S1) in a pseudocolor plot. There, it can be seen that the highest $r^2$ is found over C3 at 12 Hz. In addition, the spectrum is attenuated at this particular location and frequency during MI compared to the rest condition, so the selected features were considered to be consistent with the sensorimotor rhythm properties. Hence, the spectrum changes on C3 at 12 Hz would be a suitable feature for the training session. The feature that was used for each subject in each training session was recorded.

It should be noted that the significance of the $r^2$-value was not tested because the feature with the highest $r^2$-value was the best value that could be used for calibration because it was the feature that displayed the higher linear association between the EEG with the task, based on the $r^2$ definition [42], while it was restricted to follow the reported behavior that is expected for the EEG during a motor task. Also, it was expected that some subjects could have a feature with a non significant $r^2$-value because the experimental protocol is focused on the adaptation of the subject to the BCI during the learning process of BCI operation, so the subjects are expected to not produce a noticeable SMR pattern in every experimental session.

*Cursor Task.* The feature selected during the Calibration Analysis phase was utilized to set up the cursor task for the training session. This setup allowed the BCI to compare the current spectral measure of the chosen location and frequency against that of the previous 4 s. This comparison aimed to determine whether there was an attenuation (ERD) or an increase (ERS) in the EEG signal. This outcome was used to control a Cursor Task for 5 runs of 32 trials, with one-minute breaks between runs. In each trial, an objective was displayed for 1 s either at the upper or bottom right corner of the screen, as shown in Fig. 2 (bottom). Then, a cursor appeared at the left side of the screen and started to move at a constant rate toward the right side for 3 s. In case ERD was detected, which is expected during MI of the dominant hand, the cursor moved up on the screen. On the other hand, the cursor went down if ERS was detected, which would occur if the user was relaxed. The subject had the goal of hitting the presented objective with the cursor in each trial. If the user had success in a trial, the objective and the cursor would change its color for 1 s. At the end of each run, the percentage of hits, i.e., the accuracy, was recorded.

Regarding the EEG signal processing in this phase, the mean of C3’s neighbouring electrodes (F3, Cz and P3) was subtracted to the signal from C3 to approximate a Laplacian filter, which typically subtracts a fixed weighted sum from a subset of channels with the aim of filtering the signal components that are common to all these channels and, thus, not related to the task [45]. In addition to the spatial filter, a fourth-order bandpass Butterworth was applied to obtain the amplitude of the EEG signal component related to the user-defined frequency. For estimating the amplitude value, the signal was squared and then its square root was obtained. This was performed considering that bandpower can be estimated by bandpass filtering the signal in the required band and squaring the result [46, 47]. The cursor movement was calculated as:

3 $$\begin{aligned} x_{out}= -\frac{x_{in}-\overline{x}_{in}}{s_{in}}, \end{aligned}$$

where $x_{in}$ is the amplitude at the most recent epoch, while $\overline{x}_{in}$ and $s_{in}$ are the mean and the standard deviation of the amplitude from the previous 4 s, respectively. The numerator determines the level of change in the amplitude at the user-defined frequency and the denominator helps to regulate the movement of the cursor on the screen. This gain is relevant because the modulation ability of SMR can vary within the same session, and this modulatory effect does not appear to induce a shift in the distribution of power, but alters its variance [13]. For example, in Fig. 1 can be seen that the PSD values close to the user-defined frequency can vary. Further details of this normalizer can be found on the BCI2000 website.

### EEG processing and analysis

Once the subjects had completed their training, the variation of the selected EEG features across the training was evaluated for each user as the standard deviation of the chosen frequencies of the four sessions. This evaluation was meant to describe the stability of the features during the training process. This was carried out under the hypothesis that the selected features would be more stable for effective constant training, considering that when a stimulus is administered to affect brain activity, such as brain entrainment, the variability across features is reduced [48]. In order to determine how effective the training was, a representative accuracy was obtained for each user. To calculate this value, the mean accuracy of the five Cursor Task runs of each session was obtained and the highest accuracy from all sessions was selected, as it was the best result that the user could get with the training protocol, so it was chosen as an indicator of the potential of the subject. Next, the correlation of the standard deviation of the EEG features and the representative accuracy of the users was calculated in order to determine if there exist a relation between both variables.

After the previous analysis, the EEG signals were processed with the Multi-channel Wiener Filter (MWF) toolbox for artifact rejection [49]. This toolbox was selected because it can remove any type of artifact marked a priori, it does not require additional measurements such as the electrooculogram (EOG), and it does not necessarily require to mark the entire EEG signals that are being processed. The first characteristic was crucial because the EEG signals were not only contaminated with occasional blinking artifacts, but some of them also had cardiac artifacts that would normally exceed an amplitude of 100 $\mu$V. Then, all cardiac or eye artifacts or any component greater than 100 $\mu$V were selected manually in each run to construct a MWF to filter and clean the EEG signals.

Once the recordings were cleaned with the artifact rejection algorithm, EEG were signals of the ‘Stimulus Presentation’ runs of the first experimental session were segmented for each user into the dominant-hand MI and rest EEG fragments. To achieve this, each of the trials that involved right-hand MI was identified (i.e., the ones in which the right arrow was presented, as can be seen in Fig. 2 (top)) and the 4 s of EEG where the user performed motor imagery (right arrow) were considered the motor-imagery segment, while the previous 4 s (empty screen) where considered the rest segment of the trial. Only the last 4 s of the EEG were considered for the analysis of the rest state to obtain the same number of samples in all EEG segments for all subsequent analyses.

Then, two different processing routines were applied in MATLAB to compare the users with high and low BCI performance using these EEG signal segments: one to analyze the EEG synchronization changes for the $\theta$ (4–8 Hz), $\alpha$ (8–13 Hz), $\beta$ (13–30 Hz) and $\gamma$ (31–70 Hz) bands and another for analyzing through PDC the functional couplings between each pair of electrodes in each EEG band that may be relevant during BCI training. It should be mentioned that due to the overlapping frequencies of the $\mu$-rhythm with the $\alpha$ and presumably the $\beta$ range [7, 8], the analysis was made only in terms of these two bands. Therefore, the $\mu$-rhythm is not distinguished from them. The frequency range of each band was the same that was used for the predictor in [20] because that study described the relation of all these bands and BCI performance. Still, for a more comprehensive analysis of the $\gamma$ band, three different frequency ranges were analyzed to consider the possible variation of behaviour between the lower and higher frequencies of the $\gamma$ band: $\gamma _{low}$ (31–45 Hz), $\gamma _{high}$ (46–70 Hz) and $\gamma _{low+high}$ (31–70 Hz). The frequency that divided the low and high $\gamma$ frequencies was selected so that the ranges were relatively similar to those in other studies studies [20, 50–53], although there is variability in the gamma frequency ranges reported among studies. This was performed due to the possible variation of results that seems to occur in studies of the $\gamma$ band depending on the frequency range that is analyzed. For example, even though studies indicate that low-$\gamma$ ERD can be initially observed over the sensorimotor cortex during a motor task, followed by $\gamma$ ERS after the movement onset that occurs predominantly in the high-$\gamma$ frequencies, the response is not homogeneous between subjects and recording sites [51, 54]. Then, lower and higher $\gamma$ oscillations can show an opposite behaviour in their modulation over the sensorimotor region [54, 55] and, thus, non-homogeneus response across subjects can be exhibited because the frequency at which there is the crossover between the expected ERD and ERS can vary across subjects and recording sites [51]. Hence, it seems necessary to analyze the $\gamma$ activity in narrower frequency bands and compare with studies under similar frequency ranges.

It should be noted that only the EEG data from the ‘Stimulus Presentation’ runs of the first session were analyzed in order to determine if there were some indicators that could be related to the representative accuracy of the sessions before performing any training with the BCI system through the Cursor Task runs. The procedures for ERS/ERD and PDC analysis are described next.

#### ERS/ERD analysis

Each MI and rest segment of every trial was processed with a surface Laplacian filter to enhance the activity of each EEG sensor by attenuating the low-spatial-frequency components. In particular, the *laplacian_perrinX* function was used [56, 57]. The surface Laplacian is a spatial filter for EEG data that enhances the high spatial-frequencies (i.e., local activity) and attenuates the low spatial-frequencies (i.e, activity that is present at most electrodes) [57]. It is applied before any time-frequency analysis. There are several surface Laplacian algorithms that yield comparable results. In general, they compute the second spatial derivative of topographical activity by subtracting a weighted sum of activity across all electrodes from the activity of each electrode. Specifically, in the specific case of the Perrin function (spherical spline method), the electrode positions defining the weighting are defined by approximating the head by a sphere. The surface Laplacian mitigates volume-conduction effects and is reference independent, which makes it a good choice for connectivity analyses [57]. Thus, it represents a spatial filter that is compatible with both ERS/ERD and PDC analyses.

After the spatial filtering, the Welch’s PSD was calculated for each segment using a window size of 1 s and an overlapping window of 0.5 s. The PSD was evaluated for frequency values from 4–70 Hz in each EEG channel. Then, the ERD/ERS was estimated for each trial using a gain model based on the logarithmic power ratio (PR) [58]:

4 $$\begin{aligned} {PR}(f,c)= \log _{10} \biggl (\frac{Y_{MI}(f,c)}{Y_{rest}(f,c)}\biggr ), \end{aligned}$$

where *PR*(*f*, *c*) is the change in synchronization on channel *c* at frequency *f*, whereas $Y_{MI}(f,c)$ and $Y_{rest}(f,c)$ represent the PSD at channel *c* and frequency *f* for a MI fragment and its previous rest fragment, respectively. The gain model is common in EEG studies and it allows the normalization of the distribution, which was originally non normal. This was verified with a Lilliefors test for normal distribution, with a significance threshold of $\alpha =0.05$. Note that the logarithm in the expression indicates that if there is ERD or ERS during MI respect to the rest reference period, the value of *PR*(*f*, *c*) would be negative or positive, respectively, while it would be equal to zero dB if there was no change on the PSD for the channel and frequency that are being analyzed.

After *PR*(*f*, *c*) values were calculated, a Welch’s t-test with a significance level of $\alpha =0.05$ was used to compare the *PR*(*f*, *c*) values of all the trials of the users with high performance (representative accuracy $\ge$70%) against the subjects with low performance (<70%), i.e., beyond or below the standard level to determine that control was achieved in a two-class BCI [59]. The p-values of the comparison of *PR* values of proficient users and subjects with low BCI performance were obtained for each frequency band and each channel through the MATLAB function that performs the t-test. Multiple comparison corrections using the Benjamini-Hochberg method were performed.

It should be noted that based on the number of 32 trials per run in the experimental protocol, the accuracy threshold of 70% close to the chance level, which would be 65. 62% with a significance level of $\alpha =0.05$ for a two-class BCI. This threshold considers that the probability of each correct trial follow a binomial distribution whose confidence intervals can be calculated [60]. In MATLAB, the significance threshold *St*, can be calculated using the command $St=binoinv(1-\alpha ,n,1/c)\times 100/n$, where *n* is the number of trials, *c* the number of classes [61].

#### PDC analysis

The estimation of the PDC of the MI and rest segments of every trial and its statistical significance was carried out in MATLAB using the AsympPDC package [62], excluding the electrodes Fp1, Fp2, O1, and O2 to simplify the computational analysis. The PDC was selected to analyze functional connectivity because it cannot only describe the synchronicity between the signals from different channels, such as the direct coherence or correlation, but it can also provide information about how the analyzed brain regions are functionally connected based on the direction of their interaction (i.e., feeback or feedforward interactions) [63]. For this purpose, the PDC should evaluate if a time series can predict another time series (Granger causality), which is performed by fitting autoregressive models [64]. Note that this requires the definition of the number or prior predictions (model order), and a balance between the prediction error and the model complexity can be achieved with help of the Akaike’s Information Criterion method [65]. The autoregressive model can be used to isolate the interactions between one of the series from those due to the remaining ones to describe the PDC [63, 66]. Additionally, the model can be used to test for the nullity of the PDC based on the reported asymptotically normally distribution of the PDC when the latter is not close to zero, i.e., the Granger causality is absent. Otherwise, the normal approximation degrades [66]. The steps performed for the PDC analysis are described next.

First, the EEG fragments were detrended by subtracting the linear fit of the fragment to the data of each channel to deal to the possible non-stationarity of the signal and to estimate a consistent model of the signals for PDC analysis [67, 68]. Then, the model order was selected as the mode of the suggested order for the best multivariate auto-regressive model estimation of all segments, obtaining a model order of 15. This autoregressive model fitting was performed using ARfit package, which is linked within the AsympPDC package, based on the Akaike’s Information Criterion [69, 70]. After selecting the model order, the PDC values of all possible combinations of pairs of channels were calculated for each EEG segment of the dominant-hand MI and rest tasks, along with their PDC thresholds for a significance level of $\alpha =0.05$. Only PDC values greater than the threshold were kept for the next steps of the analysis because they are the only ones that are considered to contain signals with information on brain connectivity, as was previously mentioned. This selection can be considered as a step for checking the quality of PDC data.

The significant PDC values of the dominant-hand motor imagery and the rest conditions were compared for each pair of channels with a ranksum test ($\alpha \le 0.05$), taking into account the direction of the PDC interaction (channel A $\leftarrow$ channel B or channel B $\leftarrow$ channel A), in order to identify if motor imagery led to significant incremental or decremental PDC values compared to the rest state. The statistical tests were corrected using the Benjamini-Hochberg correction. The channels and direction of the incremental or decremental significant interactions were stored, while all interactions that did not show any significant change related to motor imagery performance were discarded from the analysis. It should be mentioned that the ranksum test was selected because the compared distributions were not normal, according to a Lilliefors test with a significance threshold of $\alpha =0.05$.

Next, the significant incremental and decremental PDC values that were present in at least 50% of subjects with high performance were stored. These final values are expected to describe the relevant PDC features related to good performance during MI-BCI training. It should be noted that the value of 50% of subjects was selected because the connectivity features vary between subjects, even though it can be expected that some of them involve the sensorimotor cortex region due to the nature of the task [15, 22]. Final results were focused on the interactions associated to the motor cortex (C3, Cz and C4), whose electrodes are considered optimal to detect motor imagery states [71].

## Results

### Accuracy and EEG features variation

**Fig. 3:**
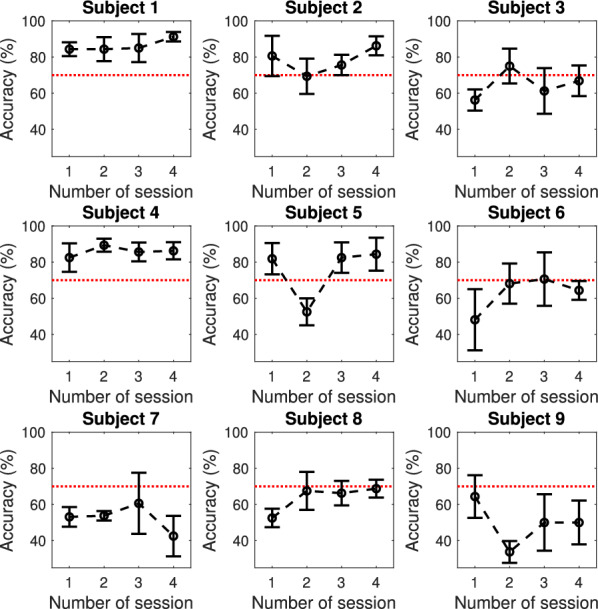
Accuracy for each session of the different subjects. Each marker and the bars represent the mean ± standard deviation of the accuracy in a particular session

Figure 3 shows for each user the mean accuracy for each of the sessions and Table 1 contains the maximum accuracy within the four sessions, which was selected as the ‘representative accuracy’ of the participant. Considering that an accuracy greater than 70% is considered as the threshold for BCI control, there were six users that had enough control in at least one session (Subjects 1, 2, 3, 4, 5 and 6), while three out of nine users (Subjects 7, 8, and 9) could not control the BCI system in any of the sessions.

**Table 1:** Representative accuracy of the subjects

Subject	Accuracy
1	91.25
2	86.25
3	75.00
4	89.38
5	84.38
6	70.63
7	60.63
8	68.75
9	64.36

**Fig. 4:**
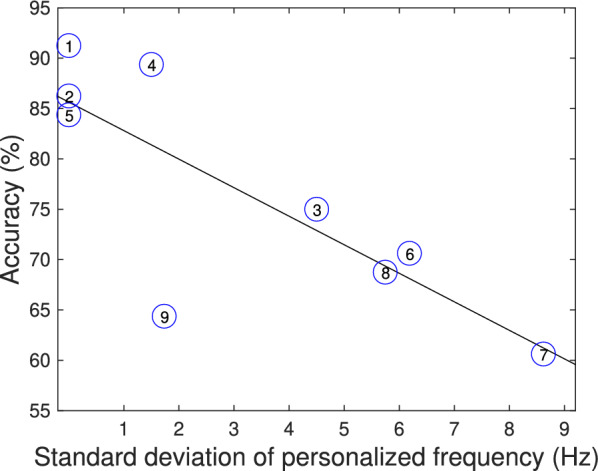
Representative accuracy for each subject and the standard deviation of the subject-defined frequencies. Each circular marker represents a subject and the line represents the linear approximation of all markers ($r=-0.80$). Subjects with higher accuracy are gathered on the left and the ones with lower accuracy are on the right, which corresponds to the lower and higher variability of the personalized frequencies that are used for BCI control across the four experimental sessions, respectively

In terms of the evolution of EEG features in relation to BCI control, Fig. 4 shows the representative accuracy and the standard deviation of the user-defined frequencies that were selected in each of the four sessions for BCI calibration, i.e., the system was configured to use the EEG at that particular frequency for controlling the cursor during the ‘Cursor Task’ in each of the experimental sessions. Each circular marker represents a subject, whose number is indicated within the marker. As can be observed, the subjects with lower representative accuracies tend to have higher variability of the EEG features that are selected according to the BCI calibration method, while the subjects with higher representative accuracy seems to have a lower variation of the user-defined frequency. Considering that the BCI calibration selects the frequency that is better discriminated within rest and motor imagery for the subject in the particular session, this means that users with higher accuracy appear to have higher consistency in the detectability of the features of their SMR, which results in a lower standard deviation. These two variables exhibit a negative correlation of $r=-0.8$.

### ERS/ERD analysis

**Fig. 5:**
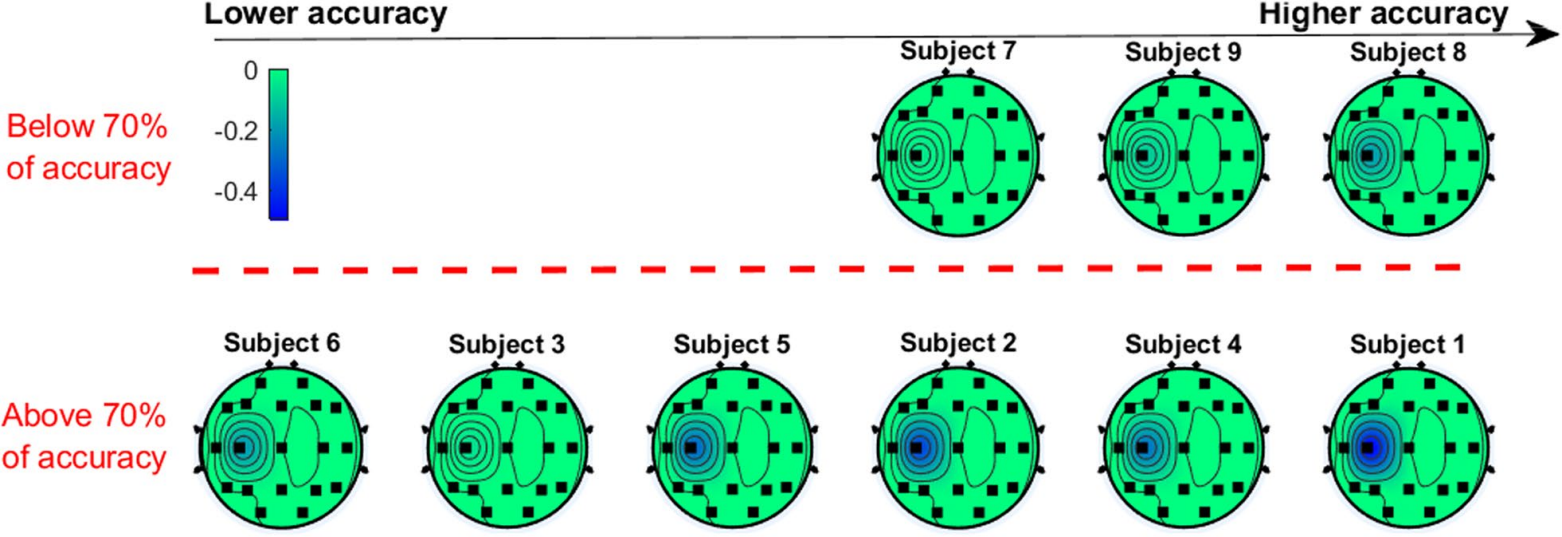
*PR* change of the users for the $\alpha$ band. The dashed line represents the threshold of 70% accuracy that is required for BCI control

Fig. 5 shows the change in synchronization (*PR*) of EEG related to motor imagery with respect to the rest state for the $\alpha$ band. In the figure, the topography of each row represents a subject and the subjects are ordered from least to greatest accuracy, according to the values from Table 1. The subjects in the top row are the ones who had an accuracy below 70%, while the ones in bottom row 70% had an accuracy above 70%. As a reference, the red dashed line represents the threshold that separates the subjects non proficient and proficient subjects. In each topography, the negative values at a particular location (blue colors on the scale of the topography) indicate the presence of significant ERD for the evaluated frequency band, while the zero values (green color) indicate that there is no significant synchronization change during motor imagery respect to the rest state. As can be seen, the only significant synchronization change is ERD over the C3 electrode. As complimentary information, Table 2 displays the summary of the significant differences that were found between the users with enough BCI control and the ones with poor BCI proficiency. The table shows the mean *PR* value for the users with an accuracy higher and lower than 70%, which are denoted as $\overline{{PR}}_{high}$ and $\overline{{PR}}_{low}$, respectively. These values represent an ERS if they are positive or ERD if they are negative and their magnitudes represent the intensity of the ERS/ERD behavior. Also, the difference between these values is included as a in terms of percentage respect to the value of $\overline{{PR}}_{low}$, as well as the p-value obtained by the Welch's t-test that compared the *PR* of the subjects from both groups.

**Table 2:** Comparison of mean *PR* values between users with higher ($\overline{{PR}}_{high}$) and lower ($\overline{{PR}}_{low}$) BCI performance

Band	Electrode	$\overline{{PR}}_{high}$	$\overline{{PR}}_{low}$	Difference (%)	p-value (corrected)
$\alpha$	C3	− 0.2861	− 0.1184	142	0.0439

As can be on the Table 2 and the Fig. 5, it seems that subjects with higher accuracy are more likely to have higher $\alpha$ ERD on C3 (i.e, the $\bar{PR}$ value is more negative), which represents a difference of 142% ($p=0.439$ .

### PDC analysis

**Fig. 6:**
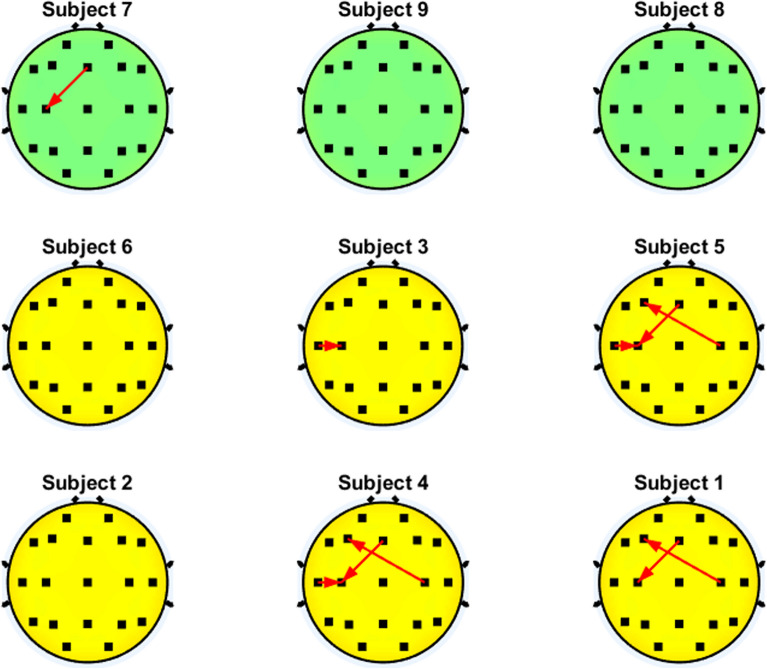
Significant interactions of the $\theta$ band that involve the motor cortex. Blue and red arrows represent the decremental and incremental PDC values related to motor imagery performance, respectively, with the inflow located at the head of the arrow

**Fig. 7:**
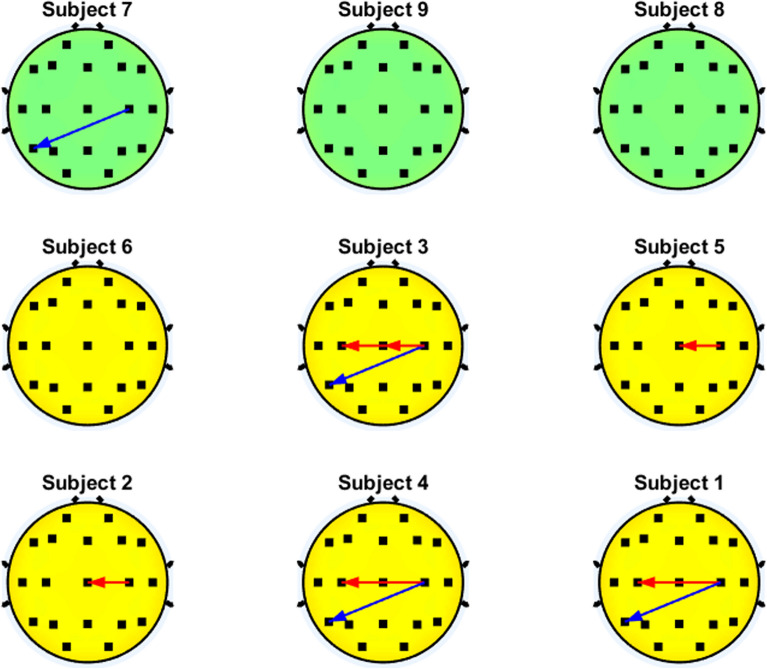
Significant interactions of the $\alpha$ band that involve the motor cortex. Blue and red arrows represent the decremental and incremental PDC values related to motor imagery performance, respectively, with the inflow located at the head of the arrow

**Fig. 8:**
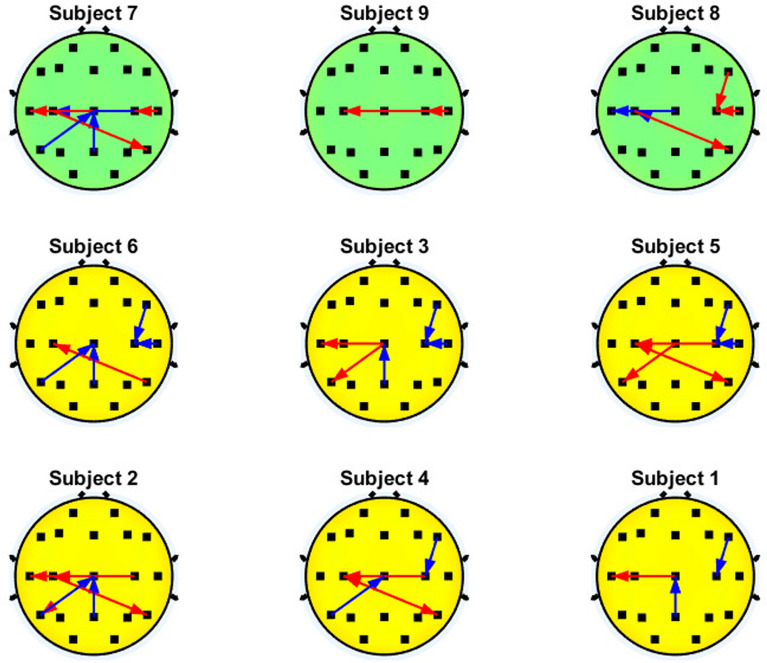
Significant interactions of the $\beta$ band that involve the motor cortex. Blue and red arrows represent the decremental and incremental PDC values related to motor imagery performance, respectively, with the inflow located at the head of the arrow

**Fig. 9:**
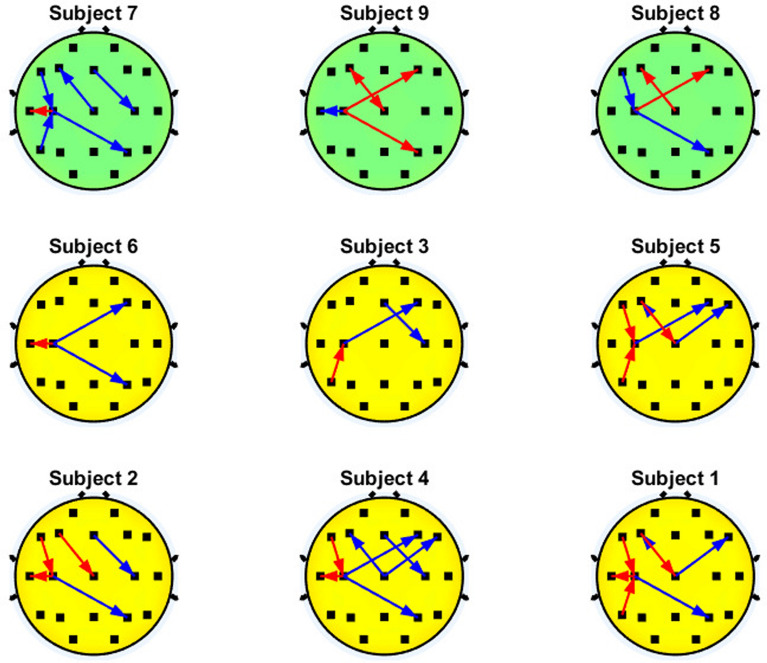
Significant interactions of the $\gamma _{low}$ band that involve the motor cortex. Blue and red arrows represent the decremental and incremental PDC values related to motor imagery performance, respectively, with the inflow located at the head of the arrow

**Fig. 10:**
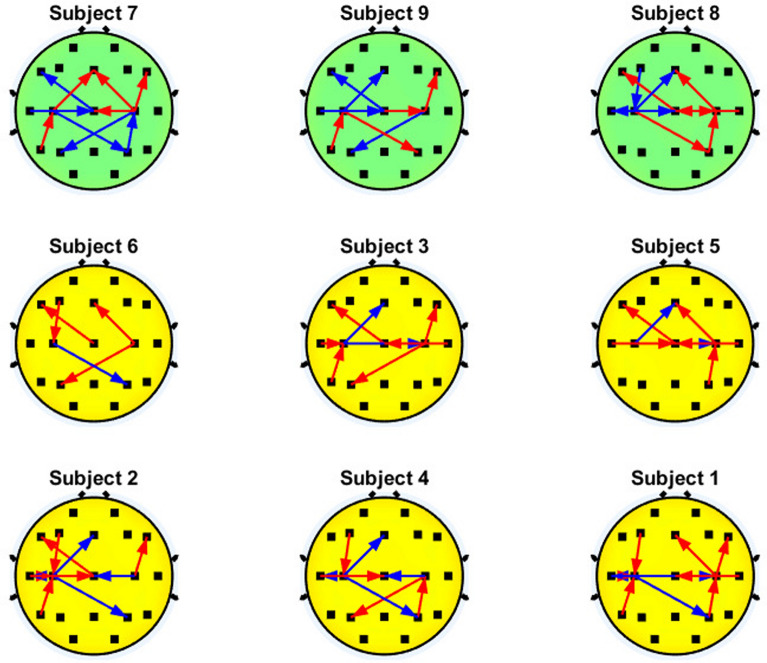
Significant interactions of the $\gamma _{high}$ band that involve the motor cortex. Blue and red arrows represent the decremental and incremental PDC values related to motor imagery performance, respectively, with the inflow located at the head of the arrow

**Fig. 11:**
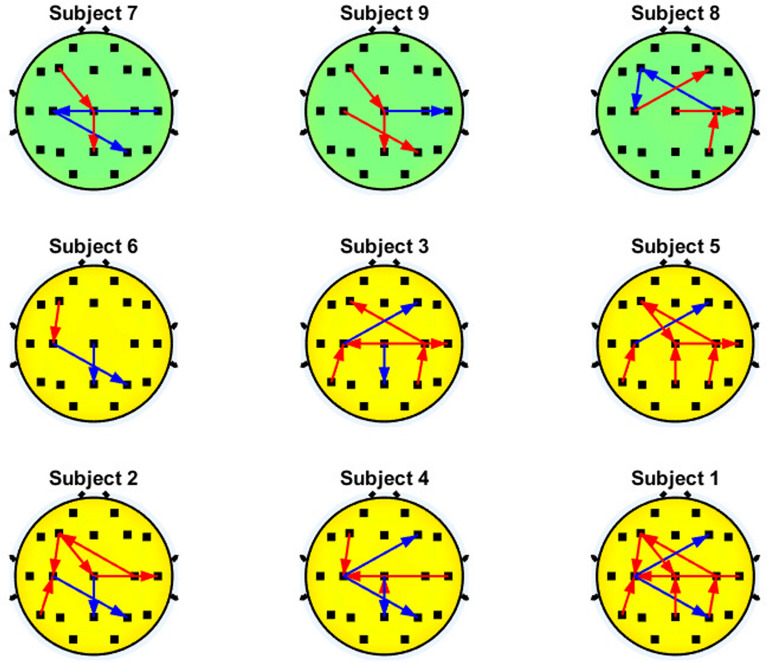
Significant interactions of the $\gamma _{low+high}$ band that involve the motor cortex. Blue and red arrows represent the decremental and incremental PDC values related to motor imagery performance, respectively, with the inflow located at the head of the arrow

Fig. 6-11 show the patterns of significant interactions that occur in the users with better performance (i.e., accuracy higher than 70%) on the motor cortex (C3,Cz and C4). Only the results related to the motor cortex were selected to allow readability of the figures. To facilitate the comparison between both groups of BCI performance, the results of the users are sorted in increasing order of their representative accuracies, which are shown in Table 1, so the user with worst accuracy can be found at the upper left side of the figure, while the user with the best accuracy is positioned at the bottom right part of the figure. In addition, subjects accuracy lower than 70% are displayed in green, otherwise they are shown in. For each subject, the interactions are displayed by using arrows that indicate the direction of the influence of brain activity between two brain regions. In case there is an attenuation of such influence during MI compared to the rest state, which means there is a diminution of functional connectivity between the two brain regions associated with the performance of MI, the arrows are shown in blue. On the other hand, if the connectivity is enhanced during MI compared to the rest state, the arrows are displayed in red.

Fig. 6 shows that most of the users with higher accuracy had either higher PDC values during motor imagery in the inflow interactions of C3 (C3$\leftarrow$Fz or C3$\leftarrow$T3) or in the outflow interactions of C4 (C4$\rightarrow$F3) on the $\theta$ band. However, several subjects with either high or low accuracy did not present any significant PDC differences between motor imagery and rest. Thus, there is no clear PDC trend in these EEG bands that can be used to differentiate users with higher or lower BCI performance.

Figure 7 shows that five out of six users with high accuracy exhibited an increment of $\alpha$ PDC values during motor imagery in the outflow interactions of C4 (C4$\rightarrow$Cz and C4$\rightarrow$C3).

Similarly to the case of the $\alpha$ band, Fig. 8 shows that three out of six subjects with higher BCI performance present an increase in the interhemispheric inflow couplings to C3 (C4$\rightarrow$C3) in the $\beta$ band, but this pattern can also be displayed by subjects with lower BCI accuracy. In addition, five out of six users with higher accuracy show a reduction of the inflow of $\beta$ couplings related to C4 during motor imagery (either C4$\leftarrow$F8, C4$\leftarrow$T4, or a combination of them), while users with poor BCI performance showed an increment of the PDC coupling in these same pairs of electrodes. Also, five out of six higher accuracy subjects and one out of three subjects show diminished inflow couplings to Cz (Cz$\leftarrow$Pz or Cz$\leftarrow$T5). This pattern seems to be shown in most participants with good performance and sometimes for the case of low performance, so it is not clear if such pattern can be related to BCI proficiency.

Figure 9 shows that users with higher accuracy tend to show a greater PDC in the $\gamma _{low}$ interactions that involve C3 in the left hemisphere (C3$\leftarrow$F7, C3$\leftarrow$T5, C3$\rightarrow$T3, or a combination of them). On the other hand, the subjects with lower performance show PDC decrements on the significant interactions during motor imagery on C3 or they can show an inconsistent PDC increments in the left hemisphere interactions, i.e, both increments (red arrows) and decrements (blue arrows) in the couplings that come and end in the left hemisphere.

In case of the $\gamma _{high}$ band, Fig. 10 shows that there exists several interactions that involve the motor cortex, but there is no clear behaviour in the interactions that help to discriminate individuals with higher or lower BCI performance.

Finally, the $\gamma _{low+high}$ band shows that the subjects with higher accuracy tend to have higher inflow interactions on C3 (C3$\leftarrow$F7, C3$\leftarrow$T5, or a combination of them), as shown in Fig. 11. Other interactions, despite being frequent, have no apparent trend to relate them to BCI control level.

## Discussion

### Accuracy and EEG features variation

Results show that six users out of nine (approximately 67%) had enough control in at least one training session, considering that an accuracy greater than 70% is considered as the threshold for BCI control and it is a typical criterion level of BCI control [12]. This percentage of 67% users in control seems to be relatively comparable with other studies that analyzed the accuracy in just one BCI training session [72, 73]. This behaviour is expected since BCI designs that are based on ERD detection show greater illiteracy compared to designs that detect evoked potentials [14]. It should be mentioned that a more recent study decreased the percentage of users without control to 14% by increasing up to 64 the number of electrodes that are available for SMR detection and through the use of the common spatial patters technique [74, 75]. However, this last study is not entirely suitable for comparison because that technique requires using more than 18 electrodes for the detection of the cognitive conditions [75], and also because the protocol of the present study focuses on producing the expected brain activity modulation on the dominant hand motor cortex as a targeted brain region during the expected learning process, instead of using the set of available electrodes to design a filter.

Regarding studies that analyze multiple BCI training sessions with feedback, it appears that there are not many studies that compare the obtained accuracy using multiple sessions. According to [11], even if machine learning techniques are applied for initial calibration and multiple sessions are performed, only up to 50% of the users are expected to exhibit moderate to high performance for asynchronous applications, based on the detectability and speed of SMR modulations. As a reference for comparison with a similar BCI design and number of sessions for a small sample of subjects, a previous study measured the accuracy for training schemes that involved the provision of different sensorial feedback modalities [35]. Each training scheme was evaluated for up to seven sessions using the BCI2000 mu-rhythm tutorial that was used as a basis for the present study. In that study, four out of six subjects (66.67%) reached a mean accuracy greater than 70% with at least one session of a particular feedback modality, taking into consideration the sessions with more than one testing run, so a mean accuracy could be calculated. Considering the similarity of this result to the one of the present study, it could be said that the percentage of users with control seem to be comparable to other studies with a similar BCI design.

In addition, results show that users with better control of the BCI system exhibit lower variation (i.e., the standard deviation) of the user-defined features that are used in calibration. This is in accordance with the proposed hypothesis in which an effective training is usually related to more stable patterns, based on results in [48]. It should be noted that users that does not show detectable EEG patterns could produce more diverse features that would result in an improper calibration, as the user-specific features could be related to brain activity changes that are not necessarily associated with the performed task. This miscalibration would result also in performance levels closer to 50%, as the feedback that is received by the user and the resulting accuracy changes could not be representative of the optimal brain activity modulation [15]. In case of the subjects that could achieve control, it may be because they can produce stable patterns since the beginning and/or they can adapt through learning to produce at will the specific patterns that are required to control the system with help of the feedback. It is known that BCIs can promote neuroplasticity, but it is important to note that many aspects of this process are still not clear [76].

It should be highlighted that the correlation value was calculated based on the variance from a sample of only 4 values (i.e., one per session). The small sample results in an inaccurate estimation of the variance [77]. Hence, results should be interpreted with caution. This limitation may be difficult to overcome with a small number of subjects, considering that extending the BCI experiments can result in a detriment on the accuracy due to the lost of motivation [78]. Thus, a strategy for keeping motivation and increasing the sample of subjects should be considered in future research.

### ERS/ERD and PDC analysis

*PR* (ERS/ERD) and PDC analyses showed that the most relevant EEG changes associated with the BCI performance were found mainly on the $\alpha$, $\beta$, $\gamma _{low}$ and $\gamma _{low+high}$ bands, in particular in relation to the contralateral and ipsilateral motor cortices. Therefore, the discussion of the results is focused on these main cases.

The analysis of the change in synchronization between the rest and right-hand MI conditions showed that participants with better BCI performance exhibit a higher $\alpha$ ERD on C3, which is the electrode associated with the right-hand motor cortex, while this high ERD was not observed on individuals with low proficiency. This $\alpha$ ERD over the contralateral motor cortex of the unimanual MI task has been reported in several studies [79, 80]. In particular, this pattern has been associated with the presence of a strong rhythm within $\alpha$ frequencies over the contralateral sensorimotor cortex during the lack of motor activity, which is desynchronized during motor imagery [18, 20].

Regarding the connectivity during motor imagery, the PDC analysis showed relevant patterns in several bands. In case of the $\alpha$ band, subjects with higher accuracy tended to show an increment of the PDC values during motor imagery in apparent interhemispheric interactions from the motor cortex that flowed out from C4 (C4$\leftarrow$Cz and C4$\leftarrow$C3). In the $\beta$ band, similar results were found (C4$\rightarrow$C3), but it seemed it could be a common pattern for all subjects, despite their BCI proficiency. The causality between the electrodes Cz, C3 and C4 has been studied in only few studies that consider the directionality of the interactions. In particular, it has been observed that the connectivity is more noticeable in the $\beta$ band than in lower frequencies like $\alpha$ [81], and both directions of coupling between C3 and C4 (C4$\leftarrow$C3 and C4$\rightarrow$C3) have been reported for the case of right-hand motor imagery in different studies [71, 81]. In the case of the interactions of Cz and C4, there appear to be also bidirectional coupling [71, 81]. The coupling of these interactions have been previously reported as part of the ones that show statistically significant changes for right-hand motor imagery, as their sensors form part of the brain regions that are part of the frontoparietal network that is involved during motor imagery tasks [82]. It must be considered that the analysis methodology of the present study does not consider the comparison of the strength of the interactions between pairs of electrodes. A more detailed analysis could be achieved if a variable related to the strength of interactions was used to determine which couplings are more viable to assess motor imagery proficiency within the BCI context.

The most stable connectivity patterns were expected to be found in the $\beta$ frequency band, considering that previous EEG-based PDC analysis showed that this specific band can provide additional information that is useful for feature selection for MI-based BCIs [83]. Results showed that users higher accuracy tend to show a reduction of the inflow of the $\beta$ couplings related to C4, while subjects with lower accuracy showed the opposite behavior. These results could indicate that the lateralization of the brain activity during MI may not be only important for ERD/ERS analysis, but also for PDC analysis. These results suggest that high MI proficiency could be related to a diminished interhemispheric sensorimotor coupling during motor imagery, which has been observed during simple unimanual motor tasks [84–87]. The interhemispheric inhibition mechanisms are thought to have a fundamental role in suppressing the mirror activity on the ipsilateral M1 cortex during unilateral hand motor tasks [86, 88–90], and they have been also observed in motor imagery tasks when the subject was specifically instructed to avoid any real movement [91, 92]. This inhibitory mechanism is part of an excitatory and inhibitory interplay between different brain regions from both hemispheres within a cortical motor network involved in programming, executing and monitoring unimanual and bimanual movements [93–98]. In particular, the ipsilateral weaker connectivity in C4 that is showed by high proficiency performance during MI (e.g., C4$\leftarrow$F8) seems to be in accordance with experiments that suggest that ipsilateral motor inhibition during unimanual tasks is mediated by both interhemispheric inhibition mechanisms and top-down modulation [99], which could allow an adequate decoupling of the motor cortical network from C4 to avoid its interference during motor imagery [100, 101]. Then, the enhanced interactions on the ipsilateral sensorimotor regions during motor imagery may hamper the performance of MI [100, 102, 103], as shown in participants with low performance. Therefore, the current study suggests that comprehension of MI-BCI performance should also encompass the analysis of PDC patterns from the ipsilateral sensorimotor cortex.

The PDC analysis also showed that users with higher accuracy tend to show greater functional connectivity in the $\gamma _{low}$ interactions that involve C3 in the left hemisphere, while the non proficient subjects show either decrements or both increments an decrements in the interactions in the same hemisphere. Following a similar pattern, subjects with higher BCI control displayed higher inflow interactions at the left hemisphere. This results seem consistent with the reported flow of activity in the brain connectome for motor imagery [6]. For the hand motor imagery, the $\gamma$ flow of activity starts over the left hemisphere when the cue to perform motor imagery is presented. Then, there is an increase of $\gamma$ outflow on several brain areas connected to motor function, particularly the primary motor area. These motor areas work in small assemblies that produce small amplitude changes in their neuronal network, but share mutual transmission among them. Then, it is reasonable to observe the interactions in C3 in this band. In addition, it has been reported that the flow of $\gamma$ occurs simultaneously to the one in $\beta$ [6], which seems in accordance with the observation of relevant results in the $\beta$ frequencies. To understand this result, it should be considered that the $\gamma$ band is fundamental for long-range sensorimotor binding and it seems that the presence of $\beta$ and $\gamma$ long-range connectivity in simple motor tasks would facilitate the sensorimotor binding that is required for performing motor tasks [53, 104]. Then, the presence of $\gamma$ activity is associated with the coupling or synchronization of distant neuronal groups and it represents an state of active information processing, which also seems to involve an $\alpha$ ERD that allows the neuronal groups to connect between each other (inhibition timing hypothesis) [40, 105].

These PDC and ERD factors that were previously described could be implemented as indicators for evaluating the difficulty of a subject for using the MI-BCI and, hence, benefiting from the neurofeedback strategy in which the system relies on. However, it would be necessary to increase the sample size to validate the results. In addition, it would be desirable the recruitment of more participants with different kind of handedness to allow the comparison between left- and right-handed users, which are reported to show a different degree of lateralization, among other differences [106]. In the long-term, this analysis could facilitate the improvement and establishment of methods for discriminating the individuals who could benefit from a neurofeedback system based on a MI-BCI.

It must be considered that all the previous results were obtained from a small sample of right-handed healthy subjects, so they should be further validated in a larger sample, specially for the case of final end-users in the case of clinical applications (e.g., patients that require motor neuro-rehabilitation), who can also be left-handed. Motor imagery based BCIs have been proposed as a tool for motor rehabilitation and, during the study of their outcomes, it has beenThe validation in target end-users is neccesary because patients can have diverse clinical and neurophysiological changes [107]. For example, in patients with an hemiparetic lesion the physiological mechanisms ($\alpha$ and $\beta$ ERD) can be transferred to the ipsilateral hemisphere [108], which indicates that there is a possible transfer of motor control from the contralateral hemisphere to the ipsilateral one [109–111].

## Conclusions

The analysis of PDC and ERS/ERD from the first session of MI-BCI training revealed crucial insights. It showed that maximum accuracy achievable with the system, after multiple sessions, hinges on the lateralization of functional connectivity. Additionally, changes in band power during motor imagery, as compared to the rest state, play a significant role. Proficient users seem to demonstrate higher $\alpha$ ERD over the contralateral sensorimotor cortex during motor imagery. Results also indicate that the $\beta$ PDC interactions should be weakened on the ipsilateral sensorimotor cortex, while the $\gamma$ ($\gamma _{high}$ and $\gamma _{low+high}$) should be enhanced on the contralateral motor cortex. This would provide additional information to the one provided in other studies that focus mainly on the PDC from the motor cortex that is contralateral to the task. These results could provide complementary features for proposing additional indicators of BCI performance, which could allow the future development of methods for assessing the possible BCI effectiveness before undergoing several training sessions with a MI-BCI. As future work, it would be necessary to increase the sample size to validate the results and recruiting more participants with different kind of handedness and patients in the long-term.

## Data Availability

The artifact-free EEG datasets generated and analysed during the current study are available in the Open Science Framework repository, in 10.17605/OSF.IO/QDS4Z. Additional data are available from the corresponding author on reasonable request.

## Abbreviations

BCI: Brain-computer interface
MI: Motor imagery
EEG: Electroencephalography
ERD: Event-related desynchronization
ERS: Event-related synchronization
SMA: Supplementary motor area
SMR: Sensorimotor
PDC: Partial directed coherence
PSD: Power spectral density
MWF: Multichannel Wiener filter
EOG: Electrooculogram
